# Innovating on Inhaled Bioequivalence: A Critical Analysis of the Current Limitations, Potential Solutions and Stakeholders of the Process

**DOI:** 10.3390/pharmaceutics13071051

**Published:** 2021-07-09

**Authors:** Jonattan Gallegos-Catalán, Zachary Warnken, Tania F. Bahamondez-Canas, Daniel Moraga-Espinoza

**Affiliations:** 1 Escuela de Química y Farmacia, Facultad de Farmacia, Universidad de Valparaíso, Valparaíso 2340000, Chile; jonattan.gallegos@labprater.cl (J.G.-C.); tania.bahamondez@uv.cl (T.F.B.-C.); 2 Via Therapeutics, Austin, TX 78712, USA; zwarnken@viatherapeutics.com; 3 Centro de Investigación Farmacopea Chilena, Universidad de Valparaíso, Valparaíso 2340000, Chile

**Keywords:** bioequivalence, inhalation, BCS, regulatory

## Abstract

Orally inhaled drug products (OIDPs) are an important group of medicines traditionally used to treat pulmonary diseases. Over the past decade, this trend has broadened, increasing their use in other conditions such as diabetes, expanding the interest in this administration route. Thus, the bioequivalence of OIDPs is more important than ever, aiming to increase access to affordable, safe and effective medicines, which translates into better public health policies. However, regulatory agencies leading the bioequivalence process are still deciding the best approach for ensuring a proposed inhalable product is bioequivalent. This lack of agreement translates into less cost-effective strategies to determine bioequivalence, discouraging innovation in this field. The Next-Generation Impactor (NGI) is an example of the slow pace at which the inhalation field evolves. The NGI was officially implemented in 2003, being the last equipment innovation for OIDP characterization. Even though it was a breakthrough in the field, it did not solve other deficiencies of the BE process such as dissolution rate analysis on physiologically relevant conditions, being the last attempt of transferring technology into the field. This review aims to reveal the steps required for innovation in the regulations defining the bioequivalence of OIDPs, elucidating the pitfalls of implementing new technologies in the current standards. To do so, we collected the opinion of experts from the literature to explain these trends, showing, for the first time, the stakeholders of the OIDP market. This review analyzes the stakeholders involved in the development, improvement and implementation of methodologies that can help assess bioequivalence between OIDPs. Additionally, it presents a list of methods potentially useful to overcome some of the current limitations of the bioequivalence standard methodologies. Finally, we review one of the most revolutionary approaches, the inhaled Biopharmaceutical Classification System (IBCs), which can help establish priorities and order in both the innovation process and in regulations for OIDPs.

## 1. Introduction

This is not a typical review that only reports a summary of information published to date. With a critical analysis of the expert opinions and conclusions collected from several authors in the literature, we have created an innovation process map that explains how technologies and methods are transferred into the bioequivalence market of orally inhaled drug products (OIDPs). This innovation path reveals the entry barriers for promising new technologies or methodologies that address current limitations related to inhaled bioequivalence.

OIDPs are an important group of medications traditionally used for the treatment of acute and chronic pulmonary affections. This is due to the fact that these diseases affect more than 200 million people and are the cause of over 3 million deaths per year worldwide [1]. Nowadays, OIDPs are a growing market for pharmaceutical companies [2]. However, from a regulatory, bioequivalence and quality control perspective, they are a difficult, complex and interesting topic of discussion.

One of the main aspirations behind bioequivalent drug products is to broaden the access to medicines to the general population by reducing their costs while maintaining an equivalent therapeutic, safety and quality profile [3]. Currently, there is an increasing interest in the inhalation route of administration, especially for drugs that are outside the classic indications for asthma and chronic obstructive pulmonary disease (COPD). Some examples of these new applications are locally acting antibiotics and vaccines, in addition to systemic indications, such as diabetes mellitus, migraine and schizophrenia [4]. Due to the complexity of OIDPs, it is a challenge to evaluate all the critical characteristics that influence their performance. This complexity in the initial product translates to difficulties in efficiently assessing their bioequivalence. The critical attributes related to the bioequivalence of OIDPs include the deposited dose of the drug to the lung, the residence time of that dose in the lung, the regional deposition of the dose across the lung and other traversed regions of the respiratory tract (tongue, throat, etc.) and dissolution of the deposited dose in the lung lining fluid. In general, regulatory agencies leading the bioequivalence evaluation are still deciding the best approaches to determine inhaled equivalence. This lack of consensus translates into less than cost-effective strategies in evaluating bioequivalence [5], discouraging innovation in the field. An example of this situation is the development of the Next-Generation Impactor (NGI), which was the last instrument officially implemented for assessing bioequivalence in 2003 [6]. Even though, at the time, the NGI was a revolutionary technology in the field, expanding the versatility of the aerodynamic characterization to dry powder inhalers (DPIs) and nebulizers, among others, it does not solve other important needs for assessing bioequivalence such as dissolution rate analysis, resulting in nearly two decades without major developments. Based on this knowledge, it is logical to ask the following: is there really an interest in improving or developing new methodologies for this process?

To understand how innovation in the inhaled bioequivalence field works, we intend to identify the stakeholders in the various realms related to bioequivalence process innovation. Additionally, an analysis of the interaction of these stakeholders is performed to assess their influence and interest in implementing new methods or technologies in the inhalation market. A list of some of these key methodologies that have been developed or can be used to determine bioequivalence is presented, with a discussion about their potential to be recognized as compendial methods.

## 2. Relationships of the Stakeholders in the Inhaled Bioequivalence Research Field

In the field of inhalation drugs, there are many groups of individuals and teams who are interested in solving unmet needs related to the bioequivalence process. Whether private or public, these groups can benefit from scientific advances in the area. However, is there really an interest in developing new methodologies? The answer can vary depending on each group, but the possibility of making the process more efficient, faster and more cost-effective is always a huge incentive for the advancement of research, medication accessibility and patient compliance. The following analysis reveals the stakeholders involved in this field, describing their motivations to promote new technologies or methods for establishing the bioequivalence of OIDPs.

### 2.1. Developers of New Bioequivalence Methodologies

These developers represent the teams of scientists and engineers from academia, industry and public institutions that work in the pharmaceutical bioequivalence field [7]. The main goal of this group of stakeholders is to promote and carry out research and development of new techniques, methodologies or protocols to compare the equivalence of a product with respect to a reference. The technologies developed by this group aim to characterize OIDPs either by their chemical or physical properties including their performance. However, complex computational-based models such as PKPD and CFD studies have been used (or even combined) to extrapolate physical and chemical data, predicting regional deposition more efficiently. In addition, these in silico prediction tools reduced the experimental cost, which is especially important in evaluating BE for OIDPs. Table 1 reflects this trend in science, where more than a third of the funded research projects rely on in silico models to achieve their goals in the research proposals. On the other hand, novel approaches to evaluate PD have recently emerged with promising strategies to overcome patient coordination during the test. Such is the case of impulse oscillometry (IOS). The IOS technique uses sound waves introduced into the patient’s airways to evaluate lung function [8]. IOS has been proposed as an endpoint sensitivity study to evaluate regional lung function changes (REF) and has been proposed as a more sensitive alternative to the traditional FEV1.

The technologies developed by this group aim to characterize OIDPs either by their chemical or physical properties, including their performance. These technologies have the potential of being used to determine bioequivalence between a reference and a test product. These technologies or methods can be either designed by the research groups previously mentioned or funded and promoted by regulatory agencies such as the U.S. Food and Drug Administration (FDA) and European Medicines Agency (EMA), which are responsible for setting standards in the inhalation field.

**Figure 1:**
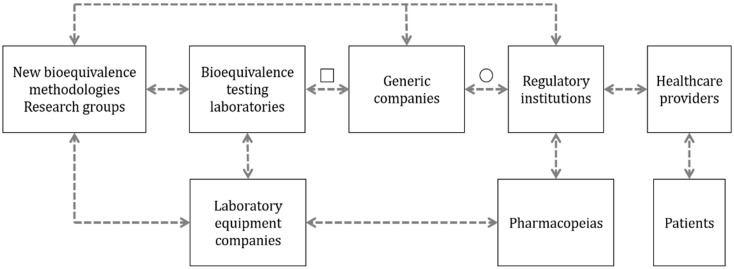
Relationship between the different stakeholders involved in the research of alternative methods with the potential to determine the bioequivalence of OIDPs. The dashed arrows describe the suggested relationship between groups. The open square represents the potential use of alternative methods in the design, development and quality control of bioequivalent OIDPs. The open circle indicates the potential for using the new alternative methods to determine the bioequivalence of new orally inhaled drug products.

The interaction between this group and bioequivalence testing laboratories is described in Figure 1. This interaction has been considered as a direct relationship based on the bioequivalence testing laboratories being the principal unit that uses the methodologies created by the developers to evaluate bioequivalence between products. Consequently, the testing laboratories are the units that identify the problems that these new methodologies are looking to solve. As an example of technology transfer between these groups, we can observe the wide case of the NGIs, a technology developed in an academic setting by Marple et al. [6], which provides essential solutions in DPI analysis and is used by BE testing laboratories [9]. The NGI simplifies the analysis of aerodynamic particle size distribution studies by facilitating sample collection, providing a method adjustable to the resistance of devices and reducing the test time [10]. In a similarly direct manner, the group of developers of new methodologies is related to companies that manufacture and sell equipment used to evaluate the performance of inhalers (Figure 1). The development of a new method and/or technology should be conducted with the intention of transferring it to the field, and as such, it should be designed to be commercially available and distributed [11]. Partnerships between methodology developers and companies with the capabilities to manufacture and distribute the technology could help to achieve the goal of technology transfer. Finally, regulatory institutions at the forefront of the BE system have a leading role as drivers of innovation. For example, regulatory agencies such as the FDA are actively looking and funding initiatives related to identifying and validating novel techniques that can be used to ease the BE process for OINDPs [12]. Some examples of these initiatives supported by the Office of Generic Drugs (OGD) are the predictive dissolution methods for OIDPs that have been evaluated using different setups for the Transwell systems, the correlation between in vivo and in vitro data when using realistic mouth-throat models and the development of a lung model that combines computational fluid dynamics (CFD) data with physiological pharmacokinetic (PBPK) modeling.

Generally, the final goal of these developers is the inclusion of the new methodology in the different pharmacopeias. Consequently, these methods would be considered a part of the compendial procedures and would be incorporated into the protocols and guidance of regulatory institutions at an international level for the development and approval of new bioequivalent OIDPs.

**Figure 2:**
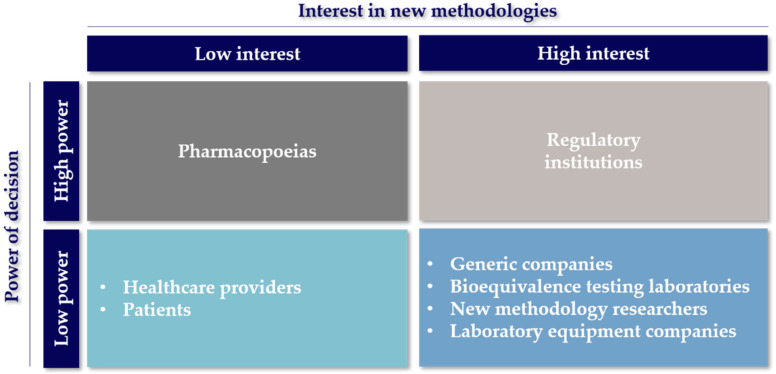
Stakeholders involved in the research of alternative methods for bioequivalence of OIDPs. Stakeholders are classified according to their interest in the development of new techniques vs. the decision making capacity and their authority to execute the decisions related to their effective use.

Finally, considering the time and resources invested in the development of new equipment, the main objective of the developers of new methodologies is for their methods to be used by other interested parties such as the producers of bioequivalent OIDPs and laboratory testing groups (Figure 2). However, they have almost no influence or decision making power to promote their ideas to “compendial” status unless their results are robust and of high quality, making them valuable for other actors in the area.

What has been described above is an extremely slow process, due to the diffusion of innovation mainly through academic journals, which may take several years to be endorsed by other scientific peers [13]. This is the case of two commercially available products for OIDP characterization [14] developed in academia, not currently recognized as compendial. The first device is the Alberta Idealized Throat (AIT) model developed in 2010 [15] by a group of academics from The University of Alberta [16] that has wide approval in the area of aerosols with more than 60 publications using the device in scientific journals. The second product is the NGI dissolution cup, developed in 2010 by McConville et al. [17] as an active part of the current discussion on issues of new methodologies to analyze OIDPs [18]. Just because these commercially available products are not compendial is not to say they are not used in regulatory submissions, and as such, obtaining compendial acceptance may not be a high priority for lab equipment companies. However, having new equipment and methods recognized as compendial would be beneficial as it would provide standardized knowledge and methods for use to assist in OIDP product development.

### 2.2. Bioequivalence Testing Laboratories

Testing laboratories focused on evaluating bioequivalence represent the contract research organizations (CRO), institutions and individuals aiming to evaluate the bioequivalence of medicines in their daily routine. Their focus is to apply existing methodologies and parameters that regulatory institutions require for approval and quality control of these inhaled medicines [19]. This group has a close relationship with the pharmaceutical companies that produce bioequivalent and generic OIDPs (Figure 1). This interaction is due to the external services that bioequivalence testing laboratories and CROs provide to the generic manufacturers, performing the bioequivalence evaluation for them [20].

The problem of bioequivalence testing laboratories arises when they are requested to perform complementary studies that are nonroutine or are not integrated into pharmacopeias. To solve this problem, they have to collaborate with companies that manufacture and sell equipment or with the developers of new methodologies to characterize OIDPs to acquire a robust and validated methodology useful for the task. In the same way, these groups also form a relationship with the laboratory equipment companies with the intention of maintaining the best technologies available in the market to study OIDPs developed in the pharmaceutical industry. Therefore, the laboratories that perform bioequivalence testing have a high interest in the development of new methods to determine the equivalence and, hopefully, the interchangeability of drug products but, at the same time, rely on other actors to carry this out (Figure 2) [21].

The limited availability of methodologies for complementary studies to determine bioequivalence is one of the main opportunities for the developers of new methodologies. This group has the potential to solve the unmet needs of this market and innovate more straightforward and cost-efficient processes than those currently available (represented as “□” in Figure 1).

### 2.3. Laboratory Equipment Companies

This group comprises companies that manufacture and/or distribute equipment used to characterize inhaled drugs. Mainly, the laboratory equipment companies sell the equipment required by regulatory agencies and pharmacopeias to characterize the OIDPs. The users of these instruments are mostly laboratories that perform new product and bioequivalence testing of OIDPs [22].

These companies have a reactive role in the bioequivalence innovation process, generally acting upon an encounter of changes or updates to monographs for the analysis of OIDPs. This translates into low relationships with other actors in the stakeholder chain, as described in Figure 1. However, when a new non-compendial technique is developed by academia or other laboratory equipment providers, there is an incentive to incorporate the technology as an available product to the companies in the bioequivalent OIDP market. An example of this situation is the sale of the AIT by a couple of companies and distributors. This well-studied device, referenced in more than 60 articles, is an alternative part of the standard pharmacopeial cascade impactors [14]. Therefore, the laboratory equipment companies in Figure 2 appear with a high interest for other methods being accepted for more commercialization, but they rely on the regulatory institutions or pharmacopeias to make them commercially attractive to invest in [23,24].

### 2.4. Generic Companies

The following group refers to the pharmaceutical industries that produce bioequivalent OIDPs for commercial purposes. For these inhaled medicines to be commercially available, they must undergo the approval process of regulatory institutions, having to meet the requirements and expectations requested by these agencies [25].

As it can be seen in Figure 1, these companies rely on the bioequivalence testing laboratories, mainly to carry out the bioequivalence studies that are required for approval. However, the companies are also involved in post-marketing studies required to ensure they maintain the quality standards over time. Some of the tests required are related to the device aerosolization performance, the aerodynamic characterization of particles and the pulmonary deposition evaluation [26,27]. The companies focused on manufacturing bioequivalent OIDPs obtained a high-interest position on the “interest vs. decision power” chart in Figure 2 as a result of the impact new methods can have on developing new bioequivalent OIDPs. Generally, new methods result in the development process being faster, more affordable and efficient. Although generic pharmaceutical companies’ influence on decision making is well positioned, the final verdict is made by the regulatory institutions [28,29].

### 2.5. Regulatory Agencies

Regulatory agencies are those public institutions whose objective is to regulate the drug products that are marketed in their jurisdiction by implementing policies that help to solve health problems that arise [30]. To do so, regulatory agencies develop guidelines and set regulations to be followed by pharmaceutical companies. The generic pharmaceutical companies must fulfill the requirements described by the regulatory agencies for the OIDPs to be approved for commercialization and be available for the treatment of diseases [30,31].

Regulatory agencies utilize a close relationship with pharmacopeias for the generation of these guidelines who evaluate the best, most robust and most reliable methodologies available for the correct analysis of inhaled medicines. An example of this interaction is the collaboration of the FDA and the United States Pharmacopeia (USP) [32] or the EMA and the European Directorate for the Quality of Medicines (EDQM) [33]. The cooperation between these entities is essential to ensure appropriate quality standards and to identify areas where an update or a new chapter in the pharmacopeia is needed [32]. One of the main objectives of developers of new methodologies for the analysis of inhaled drugs is for their methodology to be included in these guidelines. As it can be seen in Figure 1, represented as “○”, if a new method is included in the guidelines by the regulatory authority, all the stakeholders who came before the approval process will be influenced by this decision, forcing them to use the new method, instrument or technology.

Regulatory agencies have demonstrated their interest in developing new techniques for bioequivalence testing (Figure 2) through different programs to finance research [34,35], and by promoting the interaction among different stakeholders in forums and workshops for discussion [12,36,37,38,39]. Among these meetings, workshops are highlighted with events such as the “Scope and relevance of a pulmonary biopharmaceutical classification system” (Baltimore, MD, USA, 2015) [40], “New Insights for Product Development and Bioequivalence Assessments of Generic Orally Inhaled and Nasal Drug Products” (Silver Spring, MD, USA, 2018) [38] and, more recently, “Generic Drug Science and Research Initiatives” (Webcast, 2021) [41]. These workshops sponsored by the FDA, USP and scientific societies such as the American Association of Pharmaceutical Scientists (AAPS) and the International Pharmaceutical Aerosol Consortium on Regulation and Science (IPAC-RS) have covered different discussion topics from characterization and modeling to regulation, classification and future prospects around innovation on the bioequivalence evaluation of OIDPs. However, the most significant contribution of the regulatory agencies has been creating programs to support the development of new technologies for BE. For instance, the FDA, through the GDUFA division, focuses on supporting new BE research investigations based on its science and research priority initiative, which focuses on four research categories: (1) complex active ingredients, formulations or dosage forms, (2) complex routes of delivery, (3) complex drug–device combination products and (4) tools and methodologies for bioequivalence and therapeutic equivalence evaluation. These priority initiatives were considered after an agreement between the FDA, the industry and other stakeholders [42,43]. The following table summarizes the current grants and contracts awarded in the 2020 fiscal year [44]. The awarded projects clearly represent the active involvement of the FDA with the innovation of the BE of OIDPs.

The initiatives mentioned in Table 1 are funded by the GDUFA to encourage the industry and academia to participate in the BE process innovation for OIDPs. The outcome of those studies is used not just to develop new methods or technologies but also to support the assessment and approval of multiple abbreviated new drug applications (ANDAs) that intend to be recognized as generic drug products [44]. These programs generated by the FDA prove that regulatory agencies have a strong influence in the innovation chain, providing the tools to the research groups to solve the unmet needs in the complex characterization of inhalation drug products. During 2020, the GDUFA initiative generated 16 product-specific guidances (PSG) as an outcome of these research projects, an impressive contribution to the field. However, Table 1 shows just a fraction of what has been developed since 2012, when the GDUFA was created to speed up access to safe and effective generic drugs for the public [45].

### 2.6. Pharmacopoeias

For matters of this subject, pharmacopoeias refer to institutions that guarantee the optimum quality of the processes, studies, materials and standards in the pharmaceutical field. To achieve this aim, pharmacopeias publish chapters and monographs providing information about official processes, tests and methods to characterize inhalable drugs that can be used for bioequivalence evaluation [46,47].

As previously mentioned, one of the goals when developing a new emerging technology is the recognition of this technology by its inclusion in monographs or a chapter of a pharmacopoeia (see Figure 1). Therefore, the method must be accurate, effective, robust, reliable and cost-effective [48]. This knowledge and trust of materials and methods provided by pharmacopeias, together with the role they have in their collaboration with regulatory agencies, represent a great decision making power in the implementation of new methodologies for the analysis of OIDPs, as described in Figure 2.

### 2.7. Healthcare Providers

Healthcare providers are the health centers and workers in this area, such as physicians and pharmacists, who attend patients regularly. Aside from third party payer influence, they are the decision makers in the utilization of bioequivalent inhaled drugs that are available on the market. As the furthest “link” in the stakeholders’ chain, they are the ones who recommend OIDPs, prescribe OIDPs and educate patients on how to use OIDPs. They also have an important role in identifying the needs of their patients in the presence of new treatments or better access to these treatments [49].

As described by some studies, this group does not have much interest in developing new technologies for analysis of inhaled drugs, but rather, their interest lies in making OIDPs more accessible, interchangeable and of similar quality to the reference product [50]. It is important to note that, individually, physicians and pharmacists do not have much power to influence approval decisions for new bioequivalent drugs or their regulations. However, grouped in professional associations and in the face of growing health problems, they can be a great influence in these matters. As an example, we can view the innumerable efforts this group has been made in matters of COVID-19, increasing studies for treatments and drug approvals [51,52].

### 2.8. Patients

Patients are the people who are users of the medicines and treatments via inhalation prescribed and available in the market. This is the focus group of all public policies implemented by regulatory institutions to improve their life quality as OIDP users [53].

Despite not having a direct influence on the decisions regarding bioequivalence matters, all these decisions are promoted to improve the well-being of patients. Improvements in the innovation of the bioequivalence process result in user benefits, either by a greater number of drugs to choose from or by a lower price. These users are strongly influenced by the information provided by physicians about bioequivalent drugs [54] and have no major problems in switching to an equivalent product [55].

The design of new methodologies for the analysis of inhaled medicines is a concept that should be approached from as many points of view as possible—considering that a large percentage of stakeholders would benefit from a validated, accurate, reliable and robust method. Regulatory agencies such as the FDA or EMA have addressed the problem by promoting and creating instances for discussion with most of the stakeholders involved [5]. It should be observed that the EMA and FDA are the agencies most involved in these instances.

## 3. Current Regulation and Official Methodologies for Inhaled Bioequivalence

**Figure 3:**
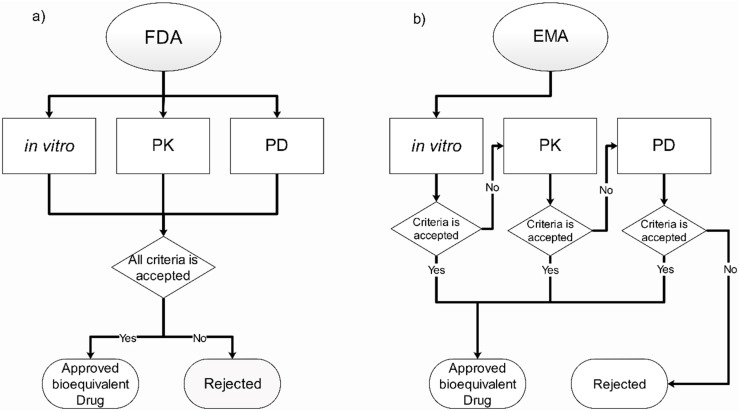
Overview and comparison of the current regulation for bioequivalence in orally inhaled drugs in the (**a**) U.S. Food and Drug Administration (FDA) and (**b**) the European Medicines Agency (EMA) [23,24,25].

This section is a non-extensive analysis of the current regulation, providing a comparison between two of the world’s leading regulatory agencies, the FDA and the EMA. The importance of their differences relates to the non-harmonization of their regulations, which, although they are very similar, are not the same (Figure 3). These discrepancies in the process end up indirectly affecting many of the stakeholders mentioned in the previous section and can finally translate into a smaller number of users reached. The latter issue is because many of the world’s regulatory agencies use the FDA and EMA regulations as a reference for their regulations.

The role of regulatory entities, as described above, is to set the conditions and requirements that pharmaceutical companies must meet for their drugs to be approved for use. To accomplish this role, different methodologies are used to evaluate the performance and efficacy of the medicines while, at the same time, comparing with the reference and verifying whether they have a therapeutic equivalence or not [56]. The guidelines for determining bioequivalence for OIDPs are available in some major regulation agencies such as the FDA [25,26] and the EMA [27]. Both have similar requirements in terms of methodologies, having categories for in vitro, pharmacokinetic (PK) and pharmacodynamic (PD) parameters. However, they do have major differences in the procedure for the bioequivalence approval. The FDA uses an approach of an aggregate weight of the evidence, which means their request results in the three categories mentioned previously. On the other hand, the EMA uses a stepwise approach based only in in vitro studies if the proposal meets all the requirements for approval; otherwise, the other categories are required [57]. This section, along with Figure 3, briefly describes the requirements needed by two of the major agencies for the approval of bioequivalent inhaled drugs.

### 3.1. In Vitro Requirements

These methods are focused on characterizing the aerodynamic distribution and the dose emitted by devices so they may be strictly adhered to through the entire device’s useful life. The officially recommended techniques are the Dosage Unit Sampling Apparatus (DUSA), cascade impactors, thin-layer chromatography plate impaction and laser light sheet photography [25,26,27]. These techniques aim to evaluate the performance of the devices, specifically on how they deliver the dose, the effective dose administered, the distribution of particle sizes and the shape of the plume in pressurized metered-dose inhaler (pMDIs) devices, respectively [58]. However, these methodologies are not without limitations. For example, while the use of fine particle doses may show an in vitro–in vivo correlation (IVIVC) for particular drugs, the use of delivered dose analyses lacks this correlation [59], as well as having an inability to analyze the angle of the plume in conditions of use of the device under an airflow that represents the patient’s breathing [60].

### 3.2. Pharmacokinetics Requirements

These studies are intended to assess the plasma drug concentration in healthy humans, evaluating PK profiles including the area under the curve (AUC) and the maximum plasma concentration (C_max_) of the drug. It is especially important to evaluate plasma concentrations for the appearance of adverse effects, establishing an equivalence in safety profiles [61]. While PK studies are often used as evidence of bioequivalence for other routes of administration, these have been particularly challenging for OIDPs. It can be difficult to evaluate plasma concentrations with many OIDPs because most of these therapies are intended to act locally [57]. Additionally, a measurement of their systemic exposure may not reflect the local PK of the drug after administration, which may be key to its efficacy and duration of action. Recently, however, Hochhaus et al. reported gathering pulmonary performance characteristics such as drug residence time and regional lung deposition from PK results with fluticasone propionate which may influence the use of PK data in bioequivalent submissions in the future [62].

### 3.3. Pharmacodynamic Requirements

Clinical dose–response-based studies assess measurable effects caused by drugs even in instances when PK analysis is not possible. There are three types of studies accepted, the bronchodilatation model, the bronchoprovocation model and clinical endpoint studies.

As an example of models used for particular drugs acting locally in the lungs, bronchodilatation models evaluate the effect of a drug when it is administrated in the airways, which is observed through an increase in the forced expiratory volume (FEV) after the usage by the patients [25,26]. These pharmacodynamic (PD) models are intended to be used with β-agonist drugs, indicated to control and prevent bronchospasm [25,26]. Similarly, corticosteroids follow the same protocols established by the FDA guidelines using FEV1 changes to assess patients’ response to the therapy.

Other types of drugs, such as corticosteroids or drugs with systemic indications, must use clinical endpoint studies. Endpoint studies are PD assessments that use a specific indicator to assess a pharmacological effect [63]. Some of these indicators include a potential change in a pharmacological response, the occurrence of an event or time elapsed without an event of interest occurring. The main limitation they have is that these parameters are less sensitive to changes in the differences in bioequivalent formulations since they depend on variable aspects of the population under study [5]. Moreover, the use of FEV1 change for corticosteroids has been challenging, considering its flat dose–response curves, which does not change significantly even after doubling the dose [64,65].

## 4. Alternative Methods for Bioequivalence

Although bioequivalence can be evaluated with the methods mentioned previously, there are still some points in the process that can be improved or complemented, either in order to increase their IVIVC and sensitivity and decrease the number of people exposed to experimentation drugs, or simply to close the gap in the knowledge for inhaled bioequivalent drugs [59]. Some of these methods that can be an alternative to demonstrate bioequivalence are described below, in addition to why they would help demonstrate bioequivalence. Additionally, the challenges that these new methods have to overcome to officially position themselves as validated methodologies will be highlighted.

### 4.1. In Vitro Methods

#### 4.1.1. Dissolution Test

Despite the fact dissolution tests are one of the fundamental tests to establish bioequivalence in most of the current pharmaceutical oral dosage forms, there is no official method for OIDPs [56]. In the case of inhaled drugs, it is challenging due to the particular physiology of the lung and the difficulties in drugs traversing the lung when delivered [66]. The low amount of liquid through the airways, approximately 10–30 mL in total spread across the large surface area of the peripheral lung [67], remains a challenge for scientists in dissolution test development, in addition to the complexity that the most recently developed formulations have obtained, such as those with prolonged release [67]. The dissolution test aims to be an in vitro parameter that can be a helpful tool to predict the in vivo behavior of formulations and, in this way, complement the discrimination of bioequivalent formulations [68].

Several authors have developed their versions or adapted dissolution tests for inhaled drugs, as shown in Table 2, including a modified USP apparatus 5, and the use of Transwells, Franz cells and flow-through cells, among other techniques, to mimic aspects of the lung dissolution environment [17,69,70,71,72,73,74]. Due to the many methods developed, there is still no consensus on which is the best approach and under what standard conditions the tests should be performed. The discussion focuses mainly on the replication of the physiological conditions of the lung to perform the test, such as the amount and type of medium, agitation and sink conditions [18,68,75]. An additional complication in the characterization of OIDPs is assessing the dissolution of the aerosolized powder under physiologically relevant conditions. The evaluation of the dissolution is accomplished using aerosol collection systems typically equipped with filters. These systems rely on the impaction of the drug to specific areas based on size which can result in the formation of agglomerates from the impactor jets, altering the dissolution of the collected powder to what would be expected in vivo [76]. Although a high IVIVC is desirable, it is not something that must be obtained. It is important to note that it is not intended to create an in vitro recreation of a lung, but rather an in vitro method that allows discriminating, in a better or, at least, a more efficient way, the equivalence between two products, compared to an in vivo method [77]. To advance the search for a standardized way to perform this analysis, the results from the different models should be related to in vivo data using statistical models with in vivo techniques. An evaluation of which of these models has a better correlation and discrimination capability should be an objective and, consequently, provide decision points to improve it.

#### 4.1.2. Mouth-Throat Models

During the inhalation maneuver, the first obstacle faced by the aerosol is the geometry of the oropharyngeal area. To assess a more representative and realistic distribution pattern of particle deposition, researchers have focused on modeling the human mouth-throat as an alternative that replaces the normal USP induction port in cascade impactors. Several authors have developed their design for a mouth-throat model (Table 2), such as the Alberta Idealized Throat (AIT) fabricated with aluminum [16], the Virginia Commonwealth University (VCU) model made with polyurethane [78], the oropharyngeal consortium model (OPC) made with polyamide [79,80] and 3D-printed models [81,82].

These models can result in differences in deposition from one another but generally have significant increases in IVIVC compared to the USP induction port [62]. Although realistic models such as OPC or VCU appear to have better performance with regard to representing the deposition in the mouth-throat than the other models, in at least one study, results from the OPC, VCU and AIT were averaged together to accommodate these differences [62,83]. From those two, the OPC model is the only mouth-throat model with clinical validation [79]. It is important to establish standard materials and sizes of the models used for these studies, as changes in these parameters can have a significant impact on the retention of particles [114]. Another important concern is the coating process for the optimal use of these models, as the particles’ bouncing and re-entrainment to the system can affect the efficacy of retention particles in the mouth-throat models [78].

#### 4.1.3. Mass-Based Plume Geometry

Similar to the mouth-throat models, the mass-based plume geometry or the Plume Induction Port Evaluator (PIPE) was developed to be an alternative to the official USP induction port for cascade impactors, but with a different objective. This device attempts to be an alternative methodology for the plume geometry analysis of pMDIs and a new tool for evaluating DPI devices [85]. The official method recommended by the USP utilizes laser sheet photography which can image a fully developed plume of the emitted aerosol. Measurements of the angles, length and width of the plume are then possible through a 2D image analysis [25]. This method has been usefully applied in the industry for years, with some disadvantages. For example, the plume is evaluated under conditions that do not represent the end use of the device by a user. Additionally, the image-based analysis can be less accurate than a chemically based analysis. If the test is performed without an airflow being applied, the deposition patterns in the mouth-throat might vary due to changes in the plume parameters [115].

PIPE is based on the USP induction port; however, it is segmented into different individual and detachable parts, as shown in Table 2. With these modifications, it is possible to chemically quantify the amount of particles retained in the different sections due to changes in the size, angle or length of the plume under controlled airflow conditions [84,85]. Although it has been developed, it still needs to correlate the changes in valves, angles and shapes of nozzles with aerosol performance, which has no way to be measured with classical methodologies [116]. This might be a good challenge for PIPE to prove its usefulness and make a change in the way the plume geometry is analyzed.

#### 4.1.4. 3D-Printed Lung Models

Determining the deposition pattern for inhaled particles is an important tool for the development and research of new OIDPs. Currently, this is only analyzed as an in vivo image-based methodology [117]. The main objective of using 3D-printed lungs for studying deposition patterns is to reduce the cost and human requirements of an in vivo study while being able to provide useful and accurate information on aerosol performance. Based on medical imaging techniques (e.g., CT scans and MRIs), a 3D replica of the lung can be built (see Table 2 for an image example) and utilized in combination with imaging methodologies or used for simulations with computational fluid dynamics (CFD) (see Section 4.3.1) to analyze the deposition patterns [87].

With the accelerated development in recent years of 3D printers, various authors have made their own lung models [86,87,88,89,90,91,92]. The main problem with these models is the complex setup for their use, making them hardly reproducible. Alongside this, there is also no consensus on which printing material is the most suitable since some have reportedly been an important influence in the retention of particles [118]. The investigations should aim to use a printer material that does not interfere with the physicochemical properties or the performance of the aerosol particles [83]. Additionally, again, researchers should remember that there is no need to replicate the complete functionality of the lung and make the setup for these methods even more complicated.

### 4.2. In Vivo Methods

#### 4.2.1. Imaging of Deposition Patterns

Even though imaging the lungs has been used for several years and been proved to be a useful method to evaluate the efficacy and characteristics of the deposition of aerosols in the lungs [94,119], it is still not accepted by the FDA as an official method to use in OIDP study [26]. There are three main imaging techniques, 2D imaging gamma scintigraphy (image represented in Table 2), single-photon emission computed tomography (SPECT) and positron emission tomography (PET) (both 3D imaging techniques). All these techniques are based on radionuclide labeling of the formulation to obtain the images, SPECT and γ-scintigraphy with technetium-99m (^99m^Tc), while PET labeling is conducted through fluorine-18 (^18^F), carbon-11 (^11^C) or nitrogen-13 (^13^N) [120].

The principal limitation of this method is the labeling process that must be conducted. This process has the potential to modify the physicochemical characteristics of the formulation and influence the results in the deposition process and no longer be representative of the original compound in study [117,121]. In the same way, the exposure to ionizing radiation increases the health risk for the workers manipulating the formulation and for the subjects in the study [97]. However, despite the limitations, imaging of deposition patterns is valuable information that can achieve relevant data for aerosol performance. Therefore, these techniques are accepted by some regulatory institutions such as the EMA for bioequivalence testing [27].

#### 4.2.2. Exhaled Nitric Oxide (eNO)

In the study of pulmonary disease, there are some biomarkers relevant for the clinical outcome of patients. A few of them can be of utility for bioequivalence in endpoint studies. For asthma disease, the concentrations of eNO are higher, which is correlated with an increased inflammatory process [122]. In the efficacy study of inhaled corticosteroids (ICS) therapy, the decrease in eNO in patients could be used as a biomarker to demonstrate equally therapeutic efficacy.

Although it has been postulated as a better and more sensitive parameter for ICS by some researchers [99,100] and has been evaluated for incorporation in the FDA’s guidance, it has also been questioned by others [123]. In this sense, the FDA sponsored a study [58] to be able to make decisions, concluding that eNO does not provide an adequate model to evaluate bioequivalence mainly because the studies were not able to establish a dose-dependent relationship with the decrease in eNO [58].

#### 4.2.3. Functional Respiratory Imaging (FRI)

As described above, the imaging technique has evolved to the point of being able to produce 3D images of the lungs and be useful for the study and development of new bioequivalent drugs. FRI is based on images obtained by computed tomography (CT) which are combined with computational fluid dynamics (CFD) (see Section 4.3.1) principles to obtain patient-specific parameters of the functionality of the lungs, which can be used as biomarkers [101]. These novel image-based biomarkers include the image-based overall resistance of the airways (see representation in Table 2), the airway volume and aerosol deposition at the peripherical airways [124] that can be effective as comparison points of bioequivalent formulations.

The limitations of the images are the same as those mentioned in the previous section, such as exposure to ionizing radiation. At the same time, it must be remembered that the functionality parameters are simulations based on these images [105]. Therefore, its representativeness of the actual functionality may not be quantitatively realistic [97]. Despite the promising results the authors have shown with this technology [101,102,103,104,105], it has not been used by different actors in academia, perhaps due to the lack of a more extensive clinical study that proves its functionality unequivocally, or the high cost of the instrumentation and patient availability.

### 4.3. In Silico Methods

#### 4.3.1. Computational Fluid Dynamics (CFD)

Based on different mathematical models, CFD can simulate airflow and particle deposition in the airways. These models integrate the aerosol characteristics, breathing patterns and airway geometries to achieve the prediction of deposition in the lung structure (see image representation in Table 2) [106,107]. Over the years, CFD has shown a good IVIVC [121], making it an important tool to facilitate and expedite the development of new inhalable drugs and their devices [108]. In the same way, this methodology has the potential to discriminate between two formulations and determine if they are bioequivalent based on their predictions of deposition in lung models.

Regarding the limitations, CFD depends mainly on the computational analytical capacity due to the complexity of the models used and the time required for them. As a consequence, there is a restriction in the amount of area that can be simulated, being limited to the upper part of the respiratory tract, more specifically from the mouth to the sixth generation of airways [109]. Furthermore, and despite their good correlation, it is necessary to validate the simulations with experimental data, which can be a problem due to the complexity of the measurement of the processes that involve the deposition of particles [97]. These limitations are expected to be solved as computer technology advances, as well as the development of new methodologies, consequently being able to compare the representativeness of the simulations with validated experimental data [109].

#### 4.3.2. Pharmacometrics

Mathematical modeling and simulations of PK parameters have been used exponentially in the pharmaceutical industry [125], although for orally inhaled drugs, this has not been the case. For the solid and liquid inhalable dosage forms, two methods have been developed, the physiology-based PK (PBPK) method and the empirical method [126]. The PBPK models use physiological parameters such as organ perfusion and physicochemical data of the formulation such as dissolution, permeability and distribution as inputs to be incorporated in a mathematical equation that models and predicts the PK [127]. This is to say that previous knowledge of the PK with the inhaled compound is required initially to be used to understand the accuracy of the prediction for BE applications. Since the PK is one of the most important aspects of bioequivalence, there must be tools that allow simulating this aspect, helping the development of new bioequivalent formulations.

As explained above, these methods have a high dependence on data from experimental (PBPK method) and clinical (empirical method) sources. The problem is the majority of this information must be produced by, typically, the generic companies for submission and is not widely available publicly. Additionally, there is a lack of validated methodologies for obtaining the key parameters such as the dissolution or pulmonary clearance of certain drugs [126]. It is for these reasons that many of the key processes in PK studies work based on assumption, but they may not be representative of what actually happens to the human body. These in silico methodologies need more investigation for establishing a correlation with in vitro and in vivo data, and a high IVIVC for the PK simulation process is very desirable [128]. However, as long as the methodologies do not advance in that sense, implementation will be a challenge following the same assumptions for critical parameters.

## 5. Future of Bioequivalence for Inhaled Drugs: Biopharmaceutical Classification System for Inhaled Medicines (iBCS)

The oral Biopharmaceutical Classification System (giBCS) is a widely accepted approach used to predict the in vivo behavior of a drug when determining the bioequivalence of immediate-release oral dosage forms. The giBCS classifies drugs into four categories based on the solubility and gastrointestinal permeability of the drug [129]. Such information is reported and accepted by different regulatory agencies as a tool that allows classifying oral drugs as candidates for a biowaiver (i.e., in vivo studies are not required for approval) [130,131].

The inhalation version of this system (iBCS) is an initiative led by Dr. Jayne Hastedt and sponsored by the Product Quality Research Institute. The group behind the iBCS initiative proposes that the same idea behind the giBCS, published by Amidon in 1995, can potentially be applied to drugs for inhalation (Table 3). In other words, it will be possible to classify drugs according to critical physicochemical parameters and lung physiology, which allows establishing a relationship between the in vitro parameters and their in vivo performance parameters [40]. The development of an iBCS can assist, guide and optimize the inhaled drug development efforts and additionally help in the bioequivalence development for inhalation, similar to the role that giBCS plays today [132].

To achieve a robust iBCS, it is necessary to fully understand the functioning of the target organ, and how the particle interaction with the tissue will determine the bioavailability or absorption of a certain drug [133]. However, it is important to consider that in this case, the bioavailability and absorption rate are not indicators of the drug effect, but indicators of the drug no longer being available in the lungs to generate a local effect. It is still necessary to clarify how these analyses can be performed in a reproducible and validated way. Therefore, the iBCS still has to overcome the challenges of a lack of validated measurement tools for the dissolution, permeability and local drug concentration of the inhaled compounds [134]. Additionally, the current iBCS only considers standard processes (dissolution and standard permeability), which is a limitation since it does not address other processes such as specific interactions with lung tissue.

The iBCS aims to be a revolutionary tool for the development and formulation of new inhaled drugs [40]. Based on the importance that the development of giBCS has for the regulatory and development aspects of oral bioequivalent drugs [135], it is normal to consider the full development of the iBCS as a useful tool in the bioequivalence of inhaled drugs. Furthermore, various are the authors that mention the projections of the iBCS in decision making on how to better address the bioequivalence of OIDPs, both from the regulatory point of view and its analysis and development [136,137,138].

## 6. Conclusions

Many stakeholders have interests in expanding the technologies available to characterize orally inhaled drug products (OIDPs), mainly to improve the process of determining bioequivalence, reduce the development time and research costs and increase the access of these drugs to the population. After analyzing the stakeholders’ opinions, the current limitations and the potential solutions for the BE process, it is quite evident that it can be improved, and the need to act to make progress towards an effective and harmonized process is highlighted. Among the stakeholders, regulatory agencies such as the EMA and the FDA stand out largely. They have led the efforts through different programs and initiatives promoting the participation, creation, development and discussion of topics of interest to improve existing methodologies [12]. In this sense, different authors from academia or private companies have developed new methodologies to complement the analysis of OIDPs, some of them with very active discussions, such as the dissolution test or in silico methods, which show a certainty to be implemented very soon.

In the methodologies that have been developed, there is one that stands out from the rest, the iBCS. This initiative is a manifestation of the interconnected work of many people from different stakeholders’ groups from the regulatory field, academy and private companies, revealing the existence of this network of stakeholders. Additionally, it demonstrates the advances that are necessary, in terms of methodologies, to progress in the improvement in the regulatory process of OIDPs, such as the need to develop a validated method for the dissolution test. The development of the iBCS must be supported so that the final product is of the highest quality and utility, and so that it can continue to support the collaborative work initiatives of different stakeholders. An idea that must be highlighted is that working together can achieve better results, and it is important to acquire the different experiences and visions that different groups of stakeholders have, looking to achieve more complete results.

## Figures and Tables

**Table 1:** Summary of the generic drug science and research initiatives related to orally inhaled drug products (OIDPs) funded by the Generic Drug User Fee Act (GDUFA) in 2020.

Objective	Grants and Contracts Related to BE of OIDPs Awarded or Completed during 2020	Institution; Grant or Contract
**Research topic 1: Drug–Device Combination Products**		
Evaluation of the impact of differences in the user interface between complex generic drug–device combination products and their RLDs on therapeutic equivalence	Development of Computational Models to Predict Delivery of Inhalation Drug Powders: From Deagglomeration in Devices to Deposition in Airways	University of Sydney; Grant (1U01FD006525)
	Patient’s Perception of DPI Airflow Resistance	Imperial College of Science and Technology, London; Contract (HHSF223201710072C)
	Comprehensive Evaluation of Formulation Effects on MDI Performance	University of Florida; Grant (5U01FD004943)
	Investigating the Impact of SMI In Vitro Characteristics on Human Airway Deposition: A Combined In Vitro/In Silico Approach	FDA
**Research topic 2: Inhalation and Nasal Products**		
Identify which factors can significantly impact how drugs are aerosolized, distributed regionally and absorbed once deposited in the lung	A Cluster-Based Assessment of Drug Delivery in Asthmatic Small Airways	University of Iowa; Grant (1U01FD005837)
	CFD and DEM Approach for Predictions of DPI Drug Delivery (U01)	Princeton University; Grant (1U01FD006514)
	Development of Computational Models to Predict Delivery of Inhalation Drug Powders: From Deagglomeration in Devices to Deposition in Airways	University of Sydney; Grant (1U01FD006525)
	Systematic evaluation of the ex-throat plume properties of MDI formulations	University of Florida; Contract (75F40119C10154C)
	Patient’s Perception of DPI Airflow Resistance	Imperial College of Science and Technology, London; Contract (HHSF223201710072C)
	Modifications and Improvements to Hybrid CFD-PBPK Models for Predication of Nasal Corticosteroid Deposition, Absorption, and Bioavailability	Applied Research Associates; Contract (75F40119C10079)
	Investigating the Microstructure of DPIs Using Orthogonal Analytical Approaches	University of Bath; Contract (HHSF223201710116C)
	Evaluating Batch to Batch Variability and Its Origins in DPIs	The University of Texas at Austin; Contract (HHSF223201810169C)
	Comprehensive Evaluation of Formulation Effects on MDI Performance	University of Florida; Grant (5U01FD004943)
	Assessment of Variability and Dose Sensitivity of FEV1 in Comparative Clinical Endpoint BE Studies of OIDPs	FDA
	CFD Models of Droplet Formulation from MDI	
	CFD Models of SMIs	
	In Vitro Performance Testing of SMIs	
	OIDP Data Collection and Analysis from Drug Product Submissions	
	Physiological Mouth-Throat Models for Inhalation Products	
	Product Quality and Performance Evaluation of Tiotropium Bromide Inhalation Powder Drug Products	
	The Use of Lung-on-a-Chip to Obtain Physiologically Relevant Parameters for OIDPs	
**Research topic 3: Locally Acting Physiologically Based Pharmacokinetic Modeling**		
Development and advance in mechanistic-based modeling, such as PBPK modeling and CFD, in order to better inform the role that product properties play in local bioavailability	A Cluster-Based Assessment of Drug Delivery in Asthmatic Small Airways	University of Iowa; Grant (1U01FD005837)
	Modeling Complex Particle Interactions in DPI Based Drug Delivery	Princeton University; Grant (1U01FD006514)
	Development of Computational Models to Predict Delivery of Inhalation Drug Powders: From Deagglomeration in Devices to Deposition in Airways	University of Sydney; Grant (1U01FD006525)
	A Multiscale Computational Framework for Bioequivalence of OIDs	CFD Research Corporation (CFDRC); Contract (HHSF223201810182C)
	CFD Models of Droplet Formulation from MDI	FDA
	CFD Models of SMIs	
	Impact of SMI In Vitro Characteristics on Human Airway Deposition: A Combined In Vitro/In Silico Approach	
	Laser Diffraction of Soft Mist Inhalers	
**Research topic 4: Quantitative Clinical Pharmacology**		
Development of clinically relevant BE criteria, design of efficient BE studies and research of alternative BE approaches. Quantitative clinical pharmacology is a quantitative platform that describes drug disposition, drug action and associated variability in humans.	Batch to Batch Variability: Exploring Solutions for Generic BE pathway	University of Maryland; Contract (75F40119C10068)
	Assessment of Variability and Dose Sensitivity of FEV1 in Comparative Clinical Endpoint BE Studies of OIDPs	FDA

RLD: reference listed drug; DPI: dry powder inhaler; MDI: metered-dose inhaler; SMI: soft mist inhaler; CFD: computational fluid dynamics; DEM: discrete element modeling; PBPK: physiologically based pharmacokinetic; FEV1: forced expiratory volume 1; OID: orally inhaled drugs.

**Table 2:** Summary of alternative methods that can be used to establish bioequivalence of inhaled drugs. The current state of the methods is based on the latest publication of the regulation, scientific journals or presentations at conferences.

Method	Key Factors for Bioequivalence	Limitations	Current State
In vitro methods			
Dissolution test [17,69,70,71,72,73,74,75]	▪ Improves understanding of the bioavailability of inhaled drugs ▪ Allows the evaluation of predictive formulation parameters in the lung environment [59]	▪ Apparatus ▪ Medium ▪ Stirring ▪ Collection of samples	Need more consensus and investigations on some topics
Mouth-throat models [16,78,79,80,81,82,83]	▪ More realistic model for human anatomy ▪ Enhanced IVIVC for cascade impactors [83]	▪ Material ▪ Size ▪ Coating	▪ Different models are commercially available ▪ Consensus is needed for the most optimal model
Quantitative plume geometry analysis [84,85]	Allows better analytical analysis of the plume geometry	Large batches for the analysis of each of the removable parts	▪ Patented method ▪ Not commercially available
3D-printed lung models [86,87,88,89,90,91,92]	Realistic in vitro deposition pattern analysis	▪ Complexity for setup ▪ Lung models ▪ Material	▪ Several models have been developed ▪ Still need more investigations
In vivo methods			
Imaging of deposition patterns [93,94,95,96,97,98]	Realistic and reliable comparisons between formulations	▪ Needs labeling of the formulations ▪ Relatively low-resolution images ▪ Exposure of humans to ionizing radiation [97]	▪ Studies are accepted at the EMA and other agencies, but they are not compulsory [27] ▪ Not accepted as bioequivalence studies at the FDA
Biomarkers: exhaled nitric oxide (eNO) [99,100]	Alternative biomarker for current endpoint studies	▪ Inclusion criteria of study subjects ▪ Influenced by patient factors ▪ Relatively low dose–response relation [58]	Not recommended for use by the FDA [5,58]
Functional respiratory imaging (FRI) [101,102,103,104,105]	Novel biomarkers for anti-inflammatory drugs	▪ Exposure of humans to ionizing radiation ▪ Image technique-based limitations	Commercially available
In silico methods			
Computational fluid dynamics (CFD) [106,107,108,109,110]	Allows simulating the flow of the device and its behavior	▪ Relies on computational capacity ▪ Limited to the 6th-generation airways ▪ Needs validation with experimental data	Commercially available software
Pharmacometrics: PBPK and empirical methods [111,112,113]	Predict the PK behavior of a formulation	▪ PBPK relies on experimental data for critical parameters such as dissolution ▪ Empirical methods depend on available clinical data	There is software available with a modality for inhalation drugs
Others			
Inhalation Biopharmaceutical Classification System (iBCS) [2,40]	▪ Categorization tool for inhaled drugs ▪ Facilitates the design of the development studies for inhaled drugs	Depends on the advances in the development of dissolution tests	Still in development

IVIVC: in vitro–in vivo correlation; PBPK: physiologically based pharmacokinetics; FDA: Food and Drug Administration.

**Table 3:** Comparison of the Biopharmaceutical Classification System for oral drugs (giBCS) with immediate-release formulations vs. the proposed categories in the Biopharmaceutical Classification System for inhaled compounds (iBCS). Adapted from Amidon et al. [118] and Hastedt et al. [123].

Class	Solubility	Permeability	IVIVC in giBCS for Oral Drugs	IVIVC in iBCS for OIDPs
I	high	high	Complete and fast absorption	Lung dose deposited is equal to the dose available for absorption Short absorption time
II	low	high	Absorption is limited to the dissolution rate	Lung dose deposited is higher than the dose available for absorption Long mean time for absorption
III	high	low	Absorption rate is limited to the intestinal permeability	Lung dose deposited is similar to the dose available for absorption Long mean time for absorption
IV	low	low	Poorly absorbed	Lung dose deposited is higher than the dose available for absorption Very long mean time for absorption

IVIVC: in vitro–in vivo correlation; giBCS: Biopharmaceutical Classification System for oral drugs; iBCS: Biopharmaceutical Classification System for inhaled drugs; OIPDs: orally inhaled drug products.

## Data Availability

Data reported in this review has been obtained primarily from the websites of the most relevant regulatory agencies in the field, and different research databases such as EBSCO, Science Direct, Pubmed, Scopus, among others.

## References

[1] World Health Organization (2020). Chronic Respiratory Diseases: Asthma.

[2] Ehrhardt C. (2017). Inhalation Biopharmaceutics: Progress towards Comprehending the Fate of Inhaled Medicines. Pharm. Res..

[3] Midha K.K., McKay G. (2009). Editorial: Bioequivalence; its history, practice, and future. AAPS J..

[4] U.S. Food and Drug Administration Approved Prescription Drugs with Therapeutic Equivalence Evaluation (Orange Book). https://www.fda.gov/drugs/drug-approvals-and-databases/approved-drug-products-therapeutic-equivalence-evaluations-orange-book.

[5] Newman B., Witzmann K. (2020). Addressing the Regulatory and Scientific Challenges with Generic Orally Inhaled Drug Products. Pharmaceut. Med..

[6] Marple V.A., Hochrainer D., Roberts D.L., Romay F.J., Miller N.C., Truman K.G., Van Oort M., Olsson B., Holroyd M.J., Mitchell J.P. (2003). Next Generation Pharmaceutical Impactor (a new impactor for pharmaceutical inhaler testing). Part I: Design. J. Aerosol Med. Depos. Clear. Eff. Lung.

[7] De Boer H. (2002). Academia in the 21st Century: An Analysis of Trends and Perspectives in Higher Education and Research.

[8] National Institute of Healthh (NIH) (2021). Grant RFA-FD-21-020: Impulse Oscillometry Endpoint Sensitivity to Regional Lung Function Changes Using Computational Fluid Dynamics (CFD) (U01) Clinical Trial Required. https://grants.nih.gov/grants/guide/rfa-files/RFA-FD-21-020.html.

[9] Marple V.A. (2004). History of Impactors—The First 110 Years. Aerosol Sci. Technol..

[10] Taki M., Marriott C., Zeng X.-M., Martin G.P. (2010). Aerodynamic deposition of combination dry powder inhaler formulations in vitro: A comparison of three impactors. Int. J. Pharm..

[11] Stevens J.M., Bagby J.W. (2001). Knowledge Transfer from Universities to Business: Returns for all Stakeholders?. Organization.

[12] U.S. Food and Drug Administration FY2016 Regulatory Science Report: Locally-Acting Orally-Inhaled and Nasal Drug Products. https://www.fda.gov/industry/generic-drug-user-fee-amendments/fy2016-regulatory-science-report-locally-acting-orally-inhaled-and-nasal-drug-products.

[13] Debackere K., Veugelers R. (2005). The role of academic technology transfer organizations in improving industry science links. Res. Policy.

[14] Copley Scientific Limited Inhaler Testing Brochure Quality Solutions for Inhaler Testing. 2019 Edition. https://www.copleyscientific.com/documents/ww/Inhaler.

[15] Finlay W.H., Golshahi L., Noga M. (2010). Choosing 3-D Mouth-Throat Dimensions: A Rational Merging of Medical Imaging and Aerodynamics. Respiratory Drug Delivery.

[16] The Aerosol Research Lab of Alberta The Alberta Idealized Throat Geometry. https://sites.ualberta.ca/~arla/alberta_idealized_throat.html.

[17] Son Y.-J., Horng M., Copley M., McConville J.T. (2010). Optimization of an In Vitro Dissolution Test Method for Inhalation Formulations. Dissolution Technol..

[18] Velaga S.P., Djuris J., Cvijic S., Rozou S., Russo P., Colombo G., Rossi A. (2018). Dry powder inhalers: An overview of the in vitro dissolution methodologies and their correlation with the biopharmaceutical aspects of the drug products. Eur. J. Pharm. Sci. Off. J. Eur. Fed. Pharm. Sci..

[19] Ocampo A., Lum S., Chow F. (2007). Current challenges for FDA-regulated bioanalytical laboratories for human (BA/BE) studies. Part I: Defining the appropriate compliance standards—application of the principles of FDA GLP and FDA GMP to bioanalytical laboratories. Qual. Assur. J..

[20] Baldeshwiler A.M. (2003). History of FDA good laboratory practices. Qual. Assur. J..

[21] Noonan P.K. (2013). Outsourcing Bioavailability and Bioequivalence Studies to Contract Research Organizations. Generic Drug Product Development and Therapeutic Equivalence.

[22] Winterhalter S., Zeschky M.B., Neumann L., Gassmann O. (2017). Business Models for Frugal Innovation in Emerging Markets: The Case of the Medical Device and Laboratory Equipment Industry. Technovation.

[23] Copley M. (2013). Improving Inhaled Product Testing: Methods for Obtaining Better In vitro-In vivo Relationships. Pharm. Technol..

[24] (2009). The Free Library Chemimage Offers Bioequivalence Technology for Drug Makers. https://www.thefreelibrary.com/chemimage+offers+bioequivlance+technology+for+drug+makers.-a0196036571.

[25] U.S. Food and Drug Administration (2016). Draft Guidance on Albuterol Sulfate. Aerosol, Metered, Inhalation. https://www.accessdata.fda.gov/drugsatfda_docs/psg/PSG_020503.pdf.

[26] U.S. Food and Drug Administration (2018). Draft Guidance on Albuterol Sulfate. Metered Powder, Inhalation. https://www.accessdata.fda.gov/drugsatfda_docs/psg/PSG_205636.pdf.

[27] European Medicines Agency (2018). Requirements for Clinical Documentation Orally Inhaled Products (OIP) Including the Requirements Demonstration of Therapeutic Equivalence between Two Use in Treatment Asthma and Chronic Obstructive Pulmonary Disease (COPD).

[28] Lexchin J., Bero L.A., Djulbegovic B., Clark O. (2003). Pharmaceutical industry sponsorship and research outcome and quality: Systematic review. Br. Med. J..

[29] DiMasi J.A., Grabowski H.G., Hansen R.W. (2016). Innovation in the pharmaceutical industry: New estimates of R&D costs. J. Health Econ..

[30] U.S. Food and Drug Administration (2018). What We Do. https://www.fda.gov/about-fda/what-we-do.

[31] European Medicines Agency (2019). From Laboratory to Patient—The Journey of a Medicine Assessed by EMA. https://www.ema.europa.eu/en/documents/other/laboratory-patient-journey-centrally-authorised-medicine_en.pdf.

[32] U.S. Pharmacopeial Convention (2018). USP and FDA Working Together to Protect Public Health|USP. https://www.usp.org/sites/default/files/usp/document/about/public-policy/USP-and-US-FDA-a-partnership.pdf.

[33] European Medicines Agency (2008). European Directorate for the Quality of Medicines and HealthCare (EDQM) of the Council of Europe. https://www.ema.europa.eu/en/partners-networks/international-activities/multilateral-coalitions-initiatives/european-directorate-quality-medicines-healthcare-edqm-council-europe.

[34] U.S. Food and Drug Administration (2016). GDUFA Reauthorization Performance Goals and Program Enhancements Fiscal Years 2018–2022. https://www.fda.gov/media/101052/download.

[35] U.S. Food and Drug Administration (2012). Generic Drug User Fee Act Program Performance Goals and Procedures. https://www.fda.gov/media/82022/download.

[36] U.S. Food and Drug Administration Leveraging Quantitative Methods and Modeling to Modernize Generic Drug Development and Review. Proceedings of the Public Workshop.

[37] DIA/FDA Conference. Proceedings of the Complex Drug-Device Generic Combination Products Meeting.

[38] U.S. Food and Drug Administration (2018). New Insights for Product Development and Bioequivalence Assessments of Generic Orally Inhaled and Nasal Drug Products. https://www.fda.gov/drugs/news-events-human-drugs/new-insights-product-development-and-bioequivalence-assessments-generic-orally-inhaled-and-nasal.

[39] Chen M.L., Blume H., Beuerle G., Davit B., Mehta M., Potthast H., Schug B., Tsang Y.C., Wedemeyer R.S., Weitschies W. (2018). The Global Bioequivalence Harmonization Initiative: Summary report for EUFEPS international conference. Eur. J. Pharm. Sci..

[40] Hastedt J.E., Bäckman P., Clark A.R., Doub W., Hickey A., Hochhaus G., Kuehl P.J., Lehr C.-M., Mauser P., McConville J. (2016). Scope and relevance of a pulmonary biopharmaceutical classification system AAPS/FDA/USP Workshop March 16–17th, 2015 in Baltimore, MD. AAPS Open.

[41] U.S. Food and Drug Administration (2021). FY 2021 Generic Drug Science and Research Initiatives Public Workshop. https://www.fda.gov/drugs/news-events-human-drugs/fy-2021-generic-drug-science-and-research-initiatives-public-workshop-06232021-06232021.

[42] U.S. Food and Drug Administration (2019). GDUFA Regulatory Science Priority Initiatives for Fiscal Year 2019. https://www.fda.gov/media/119040/download?utm_campaign=SBIA%3AFDApublishesFY2019GDUFAScienceandResearchReport&utm_medium=email&utm_source=Eloqua.

[43] U.S. Food and Drug Administration (2018). 2018 Annual Report|Office of Generic Drugs. https://fda.report/media/120593/OGD_AnnualReport_2018_ONLINE_190515_1158.pdf.

[44] U.S. Food and Drug Administration (2021). FY2020 1. U.S. Food and Drug Administration. FY2020 GDFUA Science and research report. https://www.fda.gov/media/146749/download#page=34.

[45] U.S. Food and Drug Administration (2012). GDUFA Reauthorization. https://www.fda.gov/industry/generic-drug-user-fee-amendments/gdufa-reauthorization.

[46] U.S. Pharmacopeial Convention (2019). What Is a USP Monograph. https://www.usp.org/about/public-policy/overview-of-monographs.

[47] EDQM Council of Europe Elaborations and Revisions of the European Pharmacopoeia—EDQM. https://www.edqm.eu/en/european-pharmacopoeia-elaboration-revisions-606.html.

[48] U.S. Pharmacopeial Convention (2017). Exploring Continuous Manufacturing Technology and Applications in the Pharmaceutical Industry|U.S. Pharmacopeia Blog. https://qualitymatters.usp.org/exploring-continuous-manufacturing-technology-and-applications-pharmaceutical-industry.

[49] Coulter A., Jenkinson C. (2005). European patients’ views on the responsiveness of health systems and healthcare providers. Eur. J. Public Health.

[50] Dunne S., Shannon B., Hannigan A., Dunne C., Cullen W. (2014). Physician and pharmacist perceptions of generic medicines: What they think and how they differ. Health Policy.

[51] Kupferschmidt K., Cohen J. (2020). Race to find COVID-19 treatments accelerates. Science.

[52] Webb J., Shah L.D., Lynch H.F. (2020). Ethically Allocating COVID-19 Drugs via Pre-approval Access and Emergency Use Authorization. Am. J. Bioeth..

[53] Rise M.B., Solbjør M., Lara M.C., Westerlund H., Grimstad H., Steinsbekk A. (2013). Same description, different values. How service users and providers define patient and public involvement in health care. Heal. Expect..

[54] Dunne S.S., Dunne C.P. (2015). What do people really think of generic medicines? A systematic review and critical appraisal of literature on stakeholder perceptions of generic drugs. BMC Med..

[55] Blasco Oliete M., Torres Bouza C., Medina Bustillo B., Sanz Cuesta T., Neira León M. (2003). Opinión de los usuarios de atención primaria sobre los medicamentos genéricos y el coste de la medicación. Atención Primaria.

[56] Amidon G., Lesko L., Midha K., Shah V., Hilfinger J. (2014). FDA Bioequivalence Standards.

[57] Al-Numani D., Colucci P., Ducharme M.P. (2015). Rethinking bioequivalence and equivalence requirements of orally inhaled drug products. Asian J. Pharm. Sci..

[58] Saluja B., Li B.V., Lee S.L. (2014). Bioequivalence for orally inhaled and nasal drug products. FDA Bioequivalence Standards.

[59] Forbes B., Bäckman P., Christopher D., Dolovich M., Li B.V., Morgan B. (2015). In Vitro Testing for Orally Inhaled Products: Developments in Science-Based Regulatory Approaches. AAPS J..

[60] Chambers F., De S., Baxter S., Parkinson A., Doub B., Breakwell I., Fischer M., Nagao L.M., Ag S., Group V. (2018). Plume Geometry Testing Relevance and Methodology: An IPAC-RS Survey. Respir. Drug Deliv..

[61] Kuribayashi R., Yamaguchi T., Sako H., Takishita T., Takagi K. (2017). Bioequivalence Evaluations of Generic Dry Powder Inhaler Drug Products: Similarities and Differences Between Japan, USA, and the European Union. Clin. Pharmacokinet..

[62] Hochhaus G., Chen M.-J., Kurumaddali A., Schilling U., Jiao Y., Drescher S.K., Amini E., Kandala B., Tabulov C., Shao J. (2021). Can Pharmacokinetic Studies Assess the Pulmonary Fate of Dry Powder Inhaler Formulations of Fluticasone Propionate?. AAPS J..

[63] Zou P., Yu L.X. (2014). Pharmacodynamic endpoint bioequivalence studies. AAPS Adv. Pharm. Sci. Ser..

[64] Kelly H.W. (2009). Comparison of inhaled corticosteroids: An update. Ann. Pharmacother..

[65] Raissy H.H., Kelly H.W., Harkins M., Szefler S.J. (2013). Inhaled corticosteroids in lung diseases. Am. J. Respir. Crit. Care Med..

[66] Patton J.S., Byron P.R. (2007). Inhaling medicines: Delivering drugs to the body through the lungs. Nat. Rev. Drug Discov..

[67] Smyth H.D.C., Hickey A.J. (2011). Controlled Pulmonary Drug Delivery.

[68] Forbes B., Richer N.H., Buttini F. (2015). Dissolution: A Critical Performance Characteristic of Inhaled Products?. Pulmonary Drug Delivery.

[69] Salama R.O., Traini D., Chan H.-K., Young P.M. (2008). Preparation and characterisation of controlled release co-spray dried drug–polymer microparticles for inhalation 2: Evaluation of in vitro release profiling methodologies for controlled release respiratory aerosols. Eur. J. Pharm. Biopharm..

[70] May S., Jensen B., Wolkenhauer M., Schneider M., Lehr C.M. (2012). Dissolution techniques for in vitro testing of dry powders for inhalation. Pharm. Res..

[71] Davies N.M., Feddah M.R. (2003). A novel method for assessing dissolution of aerosol inhaler products. Int. J. Pharm..

[72] Bhagwat S., Schilling U., Chen M.-J., Wei X., Delvadia R., Absar M., Saluja B., Hochhaus G. (2017). Predicting Pulmonary Pharmacokinetics from In Vitro Properties of Dry Powder Inhalers. Pharm. Res..

[73] Arora D., Shah K.A., Halquist M.S., Sakagami M. (2010). In Vitro Aqueous Fluid-Capacity-Limited Dissolution Testing of Respirable Aerosol Drug Particles Generated from Inhaler Products. Pharm. Res..

[74] Gerde P., Malmlöf M., Havsborn L., Sjöberg C.-O., Ewing P., Eirefelt S., Ekelund K. (2017). Dissolv It: An In Vitro Method for Simulating the Dissolution and Absorption of Inhaled Dry Powder Drugs in the Lungs. Assay Drug Dev. Technol..

[75] Radivojev S., Zellnitz S., Paudel A., Fröhlich E. (2019). Searching for physiologically relevant in vitro dissolution techniques for orally inhaled drugs. Int. J. Pharm..

[76] Price R., Shur J., Ganley W., Farias G., Fotaki N., Conti D.S., Delvadia R., Absar M., Saluja B., Lee S. (2020). Development of an Aerosol Dose Collection Apparatus for In Vitro Dissolution Measurements of Orally Inhaled Drug Products. AAPS J..

[77] García-Arieta A. (2014). A European Perspective on Orally Inhaled Products: In Vitro Requirements for a Biowaiver. J. Aerosol Med. Pulm. Drug Deliv..

[78] Wei X., Hindle M., Delvadia R.R., Byron P.R. (2017). In Vitro Tests for Aerosol Deposition. V: Using Realistic Testing to Estimate Variations in Aerosol Properties at the Trachea. J. Aerosol Med. Pulm. Drug Deliv..

[79] Burnell P.K.P., Asking L., Borgström L., Nichols S.C., Olsson B., Prime D., Shrubb I. (2007). Studies of the Human Oropharyngeal Airspaces Using Magnetic Resonance Imaging IV—The Oropharyngeal Retention Effect for Four Inhalation Delivery Systems. J. Aerosol Med..

[80] Olsson B., Borgström L., Lundbäck H., Svensson M. (2013). Validation of a General In Vitro Approach for Prediction of Total Lung Deposition in Healthy Adults for Pharmaceutical Inhalation Products. J. Aerosol Med. Pulm. Drug Deliv..

[81] Grgic B., Finlay W., Burnell P.K., Heenan A. (2004). In vitro intersubject and intrasubject deposition measurements in realistic mouth–throat geometries. J. Aerosol Sci..

[82] Longest P.W., Hindle M., Das Choudhuri S., Xi J. (2008). Comparison of ambient and spray aerosol deposition in a standard induction port and more realistic mouth–throat geometry. J. Aerosol Sci..

[83] Wei X., Hindle M., Kaviratna A., Huynh B.K., Delvadia R.R., Sandell D., Byron P.R. (2018). In Vitro Tests for Aerosol Deposition. VI: Realistic Testing with Different Mouth–Throat Models and In Vitro—In Vivo Correlations for a Dry Powder Inhaler, Metered Dose Inhaler, and Soft Mist Inhaler. J. Aerosol Med. Pulm. Drug Deliv..

[84] Moraga-Espinoza D., Warnken Z., Moore A., Williams R.O., Smyth H.D.C. (2018). A modified USP induction port to characterize nasal spray plume geometry and predict turbinate deposition under flow. Int. J. Pharm..

[85] Moraga-Espinoza D., Eshaghian E., Smyth H.D.C. (2018). Mass Median Plume Angle: A novel approach to characterize plume geometry in solution based pMDIs. Int. J. Pharm..

[86] Kerekes A., Veres M., Himics L., Tóth S., Czitrovszky A., Oszetzky D., Horváth A., Kugler S., Koós M., Nagy A. (2017). Determination of the deposited amount of inhalation drugs in realistic human airways by Raman and infrared spectroscopy. Measurement.

[87] Verbanck S., Ghorbaniasl G., Biddiscombe M.F., Dragojlovic D., Ricks N., Lacor C., Ilsen B., de Mey J., Schuermans D., Underwood S.R. (2016). Inhaled Aerosol Distribution in Human Airways: A Scintigraphy-Guided Study in a 3D Printed Model. J. Aerosol Med. Pulm. Drug Deliv..

[88] Kolewe E.L., Feng Y., Fromen C.A. (2020). Realizing Lobe-Specific Aerosol Targeting in a 3D-Printed In Vitro Lung Model. J. Aerosol Med. Pulm. Drug Deliv..

[89] Sonnenberg A.H., Taylor E., Mondoñedo J.R., Jawde S.B., Amin S.D., Song J., Grinstaff M.W., Suki B. (2020). Breath Hold Facilitates Targeted Deposition of Aerosolized Droplets in a 3D Printed Bifurcating Airway Tree. Ann. Biomed. Eng..

[90] Borojeni A.A.T., Noga M.L., Martin A.R., Finlay W.H. (2015). An idealized branching airway geometry that mimics average aerosol deposition in pediatric central conducting airways. J. Aerosol Sci..

[91] Lizal F., Elcner J., Hopke P.K., Jedelsky J., Jicha M. (2012). Development of a realistic human airway model. Proc. Inst. Mech. Eng. Part H J. Eng. Med..

[92] Su W.-C., Chen Y., Xi J. (2019). A new approach to estimate ultrafine particle respiratory deposition. Inhal. Toxicol..

[93] Newman S.P. (1999). Use of gamma scintigraphy to evaluate the performance of new inhalers. J. Aerosol Med..

[94] Perring S., Summers Q., Fleming J.S., Nassim M.A., Holgate S.T. (1994). A new method of quantification of the pulmonary regional distribution of aerosols using combined CT and SPECT and its application to nedocromil sodium administered by metered dose inhaler. Br. J. Radiol..

[95] Dolovich M., Labiris R. (2004). Imaging drug delivery and drug responses in the lung. Proc. Am. Thorac. Soc..

[96] Dolovich M.B., Bailey D.L. (2012). Positron emission tomography (PET) for assessing aerosol deposition of orally inhaled drug products. J. Aerosol Med. Pulm. Drug Deliv..

[97] Darquenne C., Fleming J.S., Katz I., Martin A.R., Schroeter J., Usmani O.S., Venegas J., Schmid O. (2016). Bridging the Gap Between Science and Clinical Efficacy: Physiology, Imaging, and Modeling of Aerosols in the Lung. J. Aerosol Med. Pulm. Drug Deliv..

[98] Usmani O.S., Biddiscombe M.F., Barnes P.J. (2005). Regional Lung Deposition and Bronchodilator Response as a Function of β_2_ -Agonist Particle Size. Am. J. Respir. Crit. Care Med..

[99] Hendeles L., Daley-Yates P.T., Hermann R., De Backer J., Dissanayake S., Horhota S.T. (2015). Pharmacodynamic Studies to Demonstrate Bioequivalence of Oral Inhalation Products. AAPS J..

[100] Austin D.J., Daley-Yates P.T. (2013). Evaluation Of Exhaled Nitric Oxide (eNO) As A Tool For Bioequivalence Testing Of Inhaled Corticosteroids. A60. Assessing Pulmonary Function: Airways, Mechanics, and Gas Exchange.

[101] Hajian B., De Backer J., Vos W., Van Holsbeke C., Clukers J., De Backer W. (2016). Functional respiratory imaging (FRI) for optimizing therapy development and patient care. Expert Rev. Respir. Med..

[102] De Backer J., Van Holsbeke C., Vos W., Vinchurkar S., Dorinsky P., Rebello J., Mangale M., Hajian B., De Backer W. (2016). Assessment of lung deposition and analysis of the effect of fluticasone/salmeterol hydrofluoroalkane (HFA) pressurized metered dose inhaler (pMDI) in stable persistent asthma patients using functional respiratory imaging. Expert Rev. Respir. Med..

[103] De Backer W., Vos W., Van Holsbeke C., Vinchurkar S., Claes R., Hufkens A., Parizel P.M., Bedert L., De Backer J. (2014). The effect of roflumilast in addition to LABA/LAMA/ICS treatment in COPD patients. Eur. Respir. J..

[104] Topole E., Usmani O., Mignot B., Belmans D., Van Holsbeke C., De Backer J., Osello R., Cuoghi E., Georges G., Scichilone N. (2019). Lung deposition of extrafine vs. non-extrafine triple therapies in patients with COPD using Functional Respiratory Imaging (FRI). Proceedings of the ERS International Congress 2019 Abstracts.

[105] De Backer W., De Backer J., Vos W., Verlinden I., Van Holsbeke C., Clukers J., Hajian B., Siddiqui S., Jenkins M., Reisner C. (2018). A randomized study using functional respiratory imaging to characterize bronchodilator effects of glycopyrrolate/formoterol fumarate delivered by a metered dose inhaler using co-suspension delivery technology in patients with COPD. Int. J. Chron. Obstruct. Pulmon. Dis..

[106] Oldham M.J. (2000). Computational Fluid Dynamic Predictions and Experimental Results for Particle Deposition in an Airway Model. Aerosol Sci. Technol..

[107] Nowak N., Kakade P.P., Annapragada A.V. (2003). Computational Fluid Dynamics Simulation of Airflow and Aerosol Deposition in Human Lungs. Ann. Biomed. Eng..

[108] Longest P.W., Holbrook L.T. (2012). In silico models of aerosol delivery to the respiratory tract—Development and applications. Adv. Drug Deliv. Rev..

[109] Longest P.W., Bass K., Dutta R., Rani V., Thomas M.L., El-Achwah A., Hindle M. (2019). Use of computational fluid dynamics deposition modeling in respiratory drug delivery. Expert Opin. Drug Deliv..

[110] Kleinstreuer C., Feng Y. (2013). Lung Deposition Analyses of Inhaled Toxic Aerosols in Conventional and Less Harmful Cigarette Smoke: A Review. Int. J. Environ. Res. Public Health.

[111] Simulations Plus (2019). GastroPlus PBPK Modeling Software. https://www.simulations-plus.com/software/gastroplus/.

[112] Certara USA Inc. (2019). Simcyp Simulator—Certara. Brochure. https://www.certara.com/software/physiologically-based-pharmacokinetic-modeling-and-simulation/simcyp-simulator/?ap%5B0%5D=PKPD&ap%5B1%5D=PBPK.

[113] Lukacova V., Chaudhuri S.R. (2010). Simulating Delivery of Pulmonary (and Intranasal) Aerosolised Drugs. OnDrugDelivery. http://staging.ondrugdelivery.com/wp-content/uploads/2018/11/Nov2010.pdf#page=26.

[114] Kaviratna A., Tian G., Liu X., Delvadia R., Lee S., Guo C. (2019). Evaluation of Bio-relevant Mouth-Throat Models for Characterization of Metered Dose Inhalers. AAPS PharmSciTech.

[115] Nagao L.M., Inhalation Technology Focus Group/International, Pharmaceutical Aerosol Consortium on Regulation, Sience Tests and Methods Technical Team (2002). Recommendations to the food and drug administration: Metered dose inhaler test and methods in the chemistry, manufacturing, and controls draft guidances for metered inhalers and dry powder inhalers. Drug Inf. J..

[116] Chen Y., Young P.M., Murphy S., Fletcher D.F., Long E., Lewis D., Church T., Traini D. (2017). High-Speed Laser Image Analysis of Plume Angles for Pressurised Metered Dose Inhalers: The Effect of Nozzle Geometry. AAPS PharmSciTech.

[117] Daley-Yates P.T., Parkins D.A. (2011). Establishing bioequivalence for inhaled drugs; weighing the evidence. Expert Opin. Drug Deliv..

[118] Barros A.S., Costa A., Sarmento B. (2020). Building three-dimensional lung models for studying pharmacokinetics of inhaled drugs. Adv. Drug Deliv. Rev..

[119] Newman S.P., Wilding I.R. (1998). Gamma scintigraphy: An in vivo technique for assessing the equivalence of inhaled products. Int. J. Pharm..

[120] Mobley C., Hochhaus G. (2001). Methods used to assess pulmonary deposition and absorption of drugs. Drug Discov. Today.

[121] Huang F., Zhu Q., Zhou X., Gou D., Yu J., Li R., Tong Z., Yang R. (2021). Role of CFD based in silico modelling in establishing an in vitro-in vivo correlation of aerosol deposition in the respiratory tract. Adv. Drug Deliv. Rev..

[122] Dweik R.A., Boggs P.B., Erzurum S.C., Irvin C.G., Leigh M.W., Lundberg J.O., Olin A.C., Plummer A.L., Taylor D.R. (2011). An official ATS clinical practice guideline: Interpretation of exhaled nitric oxide levels (FENO) for clinical applications. Am. J. Respir. Crit. Care Med..

[123] Gelb A.F., Moridzadeh R., Singh D.H., Fraser C., George S.C. (2012). In moderate-to-severe asthma patients monitoring exhaled nitric oxide during exacerbation is not a good predictor of spirometric response to oral corticosteroid. J. Allergy Clin. Immunol..

[124] De Backer J., Vos W., Vinchurkar S., Van Holsbeke C., Poli G., Claes R., Salgado R., De Backer W. (2014). The Effects of Extrafine Beclometasone/Formoterol (BDP/F) on Lung Function, Dyspnea, Hyperinflation, and Airway Geometry in COPD Patients: Novel Insight Using Functional Respiratory Imaging. J. Aerosol Med. Pulm. Drug Deliv..

[125] Gieschke R., Steimer J.L. (2000). Pharmacometrics: Modelling and simulation tools to improve decision making in clinical drug development. Eur. J. Drug Metab. Pharmacokinet..

[126] Kandala B., Hochhaus G., Schmidt S., Derendorf H. (2014). Pharmacometrics in Pulmonary Diseases. Applied Pharmacometrics.

[127] Bäckman P., Arora S., Couet W., Forbes B., de Kruijf W., Paudel A. (2018). Advances in experimental and mechanistic computational models to understand pulmonary exposure to inhaled drugs. Eur. J. Pharm. Sci..

[128] Borghardt J.M., Weber B., Staab A., Kloft C. (2015). Pharmacometric Models for Characterizing the Pharmacokinetics of Orally Inhaled Drugs. AAPS J..

[129] Amidon G.L., Lennernäs H., Shah V.P., Crison J.R. (1995). A Theoretical Basis for a Biopharmaceutic Drug Classification: The Correlation of in Vitro Drug Product Dissolution and in Vivo Bioavailability. Pharm. Res. Off. J. Am. Assoc. Pharm. Sci..

[130] European Medicines Agency (2020). ICH M9 Guideline on Biopharmaceutics Classification System-Based Biowaivers ICH M9 on Biopharmaceutics Classification System-Based Biowaivers. https://www.ema.europa.eu/en/ich-m9-biopharmaceutics-classification-system-based-biowaivers.

[131] U.S. Food and Drug Administration (2017). Guidance for Industry. Waiver of In Vivo Bioavailability and Bioequivalence Studies for Immediate-Release Solid Oral Dosage Forms Based on a Biopharmaceutics Classification System. https://www.gmp-compliance.org/files/guidemgr/UCM070246.pdf.

[132] Hastedt J.E. Biopharmaceutical Classification of Inhaled Medicines: Development of an iBCS. Proceedings of the 4th FDA/PQRI Conference.

[133] Eixarch H., Haltner-Ukomadu E., Beisswenger C., Bock U. (2010). Drug Delivery to the Lung: Permeability and Physicochemical Characteristics of Drugs as the Basis for a Pulmonary Biopharmaceutical Classification System (pBCS). J. Epithel. Biol. Pharmacol..

[134] Gray V. (2015). Meeting Report: AAPS Workshop on Inhalation Product Biopharmaceutical Classification System Development: Challenges and Opportunities. Dissolution Technol..

[135] Polli J.E., Abrahamsson B.S.I., Yu L.X., Amidon G.L., Baldoni J.M., Cook J.A., Fackler P., Hartauer K., Johnston G., Krill S.L. (2008). Summary workshop report: Bioequivalence, biopharmaceutics classification system, and beyond. AAPS J..

[136] Hickey A.J. (2020). Emerging trends in inhaled drug delivery. Adv. Drug Deliv. Rev..

[137] Sou T., Bergström C.A.S. (2020). Contemporary Formulation Development for Inhaled Pharmaceuticals. J. Pharm. Sci..

[138] Mercuri A., Fotaki N. (2019). In Vitro Dissolution for Inhalation Products. In Vitro Drug Release Testing of Special Dosage Forms.

